# How Do Patients and Caregivers in Advanced Illness Support One Another in Decision-Making for Patient Care? A Qualitative Interview Study of Patient and Caregiver Dyads in Specialist Palliative Care

**DOI:** 10.1089/pmr.2024.0047

**Published:** 2024-09-30

**Authors:** Norah Fagan, Andrew Davies, Geraldine Foley

**Affiliations:** ^1^ Academic Department of Palliative Medicine, Our Lady’s Hospice and Care Services, Dublin, Ireland.; ^2^ School of Medicine, Trinity College Dublin, Dublin, Ireland.; ^3^ School of Medicine, University College Dublin, Dublin, Ireland.; ^4^ Discipline of Occupational Therapy, School of Medicine, Trinity College Dublin, Dublin, Ireland.

**Keywords:** caregiver, patient, qualitative, shared decision-making, specialist palliative care, treatment decision-making

## Abstract

**Background:**

Caregivers in palliative care are tasked with supporting the patient in decision-making about treatment and care. However, how patients and their caregivers in palliative care support one another in the decision-making process is not fully understood.

**Aim:**

To decipher how patients and caregivers in specialist palliative care support one another in decision-making about patient treatment and care.

**Design:**

A qualitative study comprising semi-structured interviews. Data were thematically analyzed.

**Setting/Participants:**

Eleven patient-caregiver dyads (*n* = 22) were recruited from a large regional hospice service in Ireland providing specialist palliative care.

**Results:**

Patients and caregivers felt they supported one another in decision-making by providing emotional support and coping as a unit. Open communication coupled with an understanding of each other’s preferences helped patient-caregiver dyads navigate decision-making about the patient’s treatment and care. Patients who made decisions independent of their caregiver did so to alleviate the burden for the caregiver and because they valued having control in decision-making about their care. Trust between the patient and caregiver made patients feel able to make decisions without counsel from their caregiver. Caregivers who advocated on behalf of the patient tended to make decisions with the patient. Shared decision-making comprised patient and caregiver making decisions together as a team with the opportunity to collectively re-examine and adjust their preferences for treatment and care.

**Conclusions:**

This study identified that patients and caregivers in specialist palliative care can be emotionally supportive of one another in situations where they make decisions together about care and in situations where the patient makes decisions about care independent of their caregiver. These findings are relevant for health care professionals in palliative care who seek to promote emotional support between the patient and caregiver in discussions about treatment and care.

## Introduction

Patients in receipt of palliative care face increasingly complex decisions about treatment and care as they advance in their illness and experience symptom burden.^1–3^ Patients with advanced illness also become increasingly reliant on informal caregivers (in many cases, family caregivers) for multiple forms of support (i.e., physical, emotional, social, and financial).^4,5^ Caregivers are involved in the organization and delivery of care to patients, generally operating in an unpaid capacity and in addition to formal palliative care services.^6^ People who assume an informal caregiver role are often tasked along with health care professionals with supporting the patient in the decision-making process for treatment and care.^7^ Although caregivers provide a mix of physical, psychological, and social support to their ill family members, patients with advanced illness have demonstrated the capacity to reciprocate in nonphysical domains of supportive care.^8^ There is evidence that patients and caregivers in specialist palliative care reciprocated in emotional support of one another^8^ and accommodated each other’s preferences for care out of concern for one another.^9^ Patients and caregivers in specialist palliative care also communicated openly with one another when they did not feel restricted by their reliance on their caregiver and when their caregiver assumed a caregiving role by choice rather than out of obligation.^10^ However, beyond the above, it is not fully clear how patients with advanced illness and their caregiver support one another when faced with decisions about patient treatment and care. Frameworks for decision-making and relationship functioning between the patient and caregiver in life-limiting illness^11–13^ have not necessarily situated the patient in a supportive role. In palliative care, the focus to date has been primarily on how health care professionals assisted patients and caregivers in making decisions about patient treatment and care^14^ and what was important to caregivers when they assisted patients in decision-making.^15,16^

Specialist palliative care is provided by health care professionals whose core activity is providing this care. We aimed to decipher how patients and caregivers in specialist palliative care support one another when making decisions about patient treatment and care.

## Methods

### Study design

We used a qualitative design for this study incorporating thematic analysis.^17,18^ A qualitative design was appropriate because we were primarily inductive in our approach to investigating a poorly understood phenomenon.^19^ We sought to interpret^20^ patient and caregiver accounts of their interactions and behaviors toward one another in decision-making. This study was approved by the affiliated university research ethics committee and hospice research ethics committee. All participants provided written informed consent to participate.

### Setting

The study setting was a large regional specialist palliative care service in Ireland which comprises three hospice sites. The regional service provides specialist palliative care to a catchment area of approximately 1 million people (circa 20% of the national population). It comprises a mix of inpatient and outpatient hospice care, community-based palliative care, and consultation to linked hospital (acute) care. Palliative care is provided by specialist interdisciplinary teams.

### Participant recruitment and sampling

Between November 2023 and March 2024, we recruited 11 patient and caregiver dyads (*n* = 22) across the study setting. We devised a joint patient and caregiver study invitation letter, and separate patient and caregiver information leaflets for the study. Patient and caregiver dyads across the regional palliative care service and who met inclusion criteria were then approached by hospice staff who were separate from the research team and who were not involved in patients’ care. Inclusion criteria for patients were that they were ≥18 years old, in receipt of specialist palliative care, and at an advanced stage of their illness. Advanced illness was defined as having a prognosis of six months or less. The inclusion criteria for caregivers were that they were primary caregivers to the patient and were ≥18 years old. We purposively sampled participants that met inclusion criteria for variation in contexts including, for example, patient age and life stage, and the nature of relations for each dyad. Patients' ages ranged between 39 and 93 years (mean age: 69.7 years). Eight of the 11 patients were women, and six of the 11 caregivers were men. Six of the dyads were spousal/partner dyads. For four dyads, the caregiver was an adult child of the patient, and for the remaining dyad, the caregiver was a parent of the patient. All but one of the patients had advanced cancer. Table 1 details a full summary of participants including specialist palliative care accessed by the patient and the variation in length of time since patients had first accessed specialist palliative care.

**Table 1.** Summary of Participants

Dyads	Diagnosis	Geography	Gender	Patient age (years)	Caregiver relation	Time known to SPC	Experience of SPC
P1 CG1	Cancer (Malignant)	Urban Urban	W M	89	Spouse	12 months	Community-based care & acute (hospital) care
P2 CG2	Cancer (Malignant)	Suburban Suburban	M W	88	Daughter	<1 month	Inpatient hospice care & acute (hospital) care
P3 CG3	Cancer (Malignant)	Urban Urban	W M	74	Spouse	1–2 years	Community-based care & acute (hospital) care
P4 CG4	Cancer (Malignant)	Urban Urban	M M	39	Partner	1–2 years	Community-based care, acute (hospital) care & respite care
P5 CG5	Cancer (Malignant)	Suburban Suburban	W W	54	Mother	1–2 years	Community-based care & inpatient hospice care
P6 CG6	Cancer (Malignant)	Urban Urban	W W	57	Daughter	12 months	Community-based care & acute (hospital) care
P7 CG7	Neurodegenerative	Suburban Suburban	W M	77	Spouse	1–2 months	Community-based care & acute (hospital) care
P8 CG8	Cancer (Malignant)	Suburban Suburban	W W	69	Daughter	<1 month	Community-based care & acute (hospital) care
P9 CG9	Cancer (Malignant)	Suburban Suburban	W M	69	Spouse	1–2 months	Community-based care & acute (hospital) care
P10 CG10	Cancer (Malignant)	Suburban Suburban	W M	58	Spouse	6 months	Community-based care & acute (hospital) care
P11 CG11	Cancer (Malignant)	Urban Urban	M W	93	Daughter	6 months	Community-based care & hospice outpatients/day services

W, Woman; M, Man; SPC, Specialist Palliative Care.

### Data collection

Data collection comprised semi-structured interviews with participants. Interview guides for data collection were formulated in line with the aim of the research and informed by systematic reviews conducted with colleagues focused on mutual support between patients and family caregivers in palliative care^21^ and on concordance and discordance in decision-making between patients and caregivers in palliative care.^3^ The interview schedules were compiled jointly by the authors. Questions were formulated to decipher how patients made decisions about their care (i.e., with or without their caregiver), and how the patient and caregiver supported one another in decision-making. Please see the interview schedules used for this study in Supplementary Data S1. Our preference was to interview the patient and caregiver of the dyad separately, but we anticipated that some dyads might have a preference to be interviewed together. Each dyad was given the option to be interviewed together or separately. Participants of three of the 11 dyads requested to be interviewed with their respective other. Each participant was interviewed on one occasion (whether alone or together). In total, 19 interviews were conducted with participants (three interviews from three dyads and 16 separate interviews from eight dyads). Nine dyads were interviewed in their home and two dyads were interviewed in a hospice. The interviews were on average 30 minutes in duration. All interviews were in person, digitally recorded, transcribed verbatim, and then pseudonymized in storage.

Participant member checking^22^ was performed by confirming interpretation with participants both during and at the end of interviews. Field notes were recorded by the interviewer to aid analysis of the transcribed interviews. The field notes comprised detailed observations from each interview (e.g., the interview setting and the tone and mood of participants) which helped to further contextualize the data for analysis.

### Data analysis

The data were analyzed using Braun and Clarke’s framework for thematic analysis.^17,18^ The first step involved full familiarization with the data by reading the transcripts multiple times. Initial codes were then generated across the full set of transcripts and each code was labeled in a manner that described the data coded to it.^17,18^ The data were coded for incidents, behaviors, and interactions noted in participants’ responses to the questions asked about them. Similar initial codes were then collated to form tentative themes that represented the data under them.^17,18^ The analysis was then refocused at the theme level to review how different codes combined to form a theme and to ensure that themes were fully representative of their data.^17,18^ The final stage of analysis constituted a full description and refinement of each theme.^17,18^ Microsoft Excel was used as a tool to organize the data during coding. The rigor in the analysis was ensured by the third author’s examination of all coded data and by agreement between the first and third authors on the aggregation of codes to themes and on the final description and refinement of each theme.

## Results

We detailed key findings from our study which described how patients and caregivers supported one another in decision-making for patient treatment and care. In the subsequent sections of the results, we have presented findings categorized into three overarching themes derived from the data. The first theme depicted supportive dimensions of the patient and caregiver relationship as they navigated decision-making. The remaining two themes outlined two different approaches to decision-making identified in the data in the context of supportive relationships. All participants’ quotes used to illustrate key findings were pseudonymized using ID codes as listed in Table 1 (i.e., P1 and CG1 refer respectively to the patient and caregiver of dyad no. 1).

### Navigating decision-making

Patient and caregiver support of one another in decision-making was found to be primarily emotional support. Emotional support as a form of support was evident in all participants’ responses. Emotional support comprised the ability to reassure one another and the sensitivity to reciprocate in this form of support. For example, a patient and their caregiver communicated:

He [caregiver] reassures, he gives me good advice. I want someone to bash things out with me so that I have a fifty-fifty answer in my head. I have 50% and [caregiver] will have the other 50%. So that is the way we do it. (P4)

We need emotional balance. I can tell when it [emotional support] is needed to give [but] I can be on the downside as well and this leads to emotional support from [patient]. (CG4)

Participants felt that emotional support involved coping as a unit with a life-limiting illness. Subsequently, coping as a unit allowed them to best navigate decision-making about patient treatment and care. For example, a patient of an older spousal dyad shared:

I think we are coping together and coping well. We have come to terms with the fact that we are not going to last very long [expects caregiver to die in near future due to old age] anyway and so we have to be pragmatic about decisions for me. (P1)

Coping as a unit also prompted caregivers to reflect not only on their current caregiving role but also on the patient’s future care needs and how they felt they could best accommodate and prioritize the patient’s needs. A caregiver explained:

What bothers me is further down the line as [patient] deteriorates, what my future is. But I won’t let my situation affect [patient’s] requirements. What she wants she gets. (CG7)

Efforts to be mutually supportive despite the patient’s deterioration in their health helped patients and their caregivers resolve differences in their preferences for patient care. For example, a patient and caregiver who differed in their preference for patient continuation of chemotherapy disclosed:

Initially she [caregiver] wanted me to do more chemo[therapy]. I didn’t want to do it [so] I had to counsel her why I didn’t want to do it. (P6)

After she had the chemo[therapy], it didn’t go well with her and she suffered and I saw that and when she said no, I understood. I don’t support that she stopped but I understand because I saw her suffering. (CG6)

Overall, open communication coupled with an understanding of each other’s preferences for care helped both patients and caregivers navigate decision-making.

### Independent decision-making

Some patients (*n* = 5) perceived themselves to be self-reliant and made decisions without their caregivers to negate the possibility of becoming a burden on their caregivers. For example, patients who wished to avoid placing undue burden on their respective caregivers stated:

I am kind of independent. I don’t like burdening other people with my problems. I knew my wife was starting the early stages of dementia and would not be able to mind me at home … and the fact that [daughter, CG2] has a job and her own family to run. I made the decision myself to be transferred here [hospice] … If I was at home my wife and daughter would not be able to cope … It would not be fair on them. (P2)

[In terms of the decision to come into the hospice] I [came in] as soon as I got the diagnosis, and I just told my parents what I wanted … my decision was to help make things easier for them. (P5)

Patients who reported a tendency to make decisions independent of their caregivers valued the control they felt when making decisions about their treatment and care. For example, a patient who made the decision independent of her spouse to refuse chemotherapy, and who felt supported by her spouse to do so, commented:

Being in control is important … As I say when my mind is made up, my mind is made up. At the end of the day, this is my body, my decision. (P10)

In some instances, a decision about the mode of care caused conflict when a patient decided about an aspect of their care without counsel from their caregiver. In these situations, conflict arose when the patient declined formal health care support and when their caregiver felt that such support was fully necessary. For example, participant CG6 reported:

With the care that she [patient] has been granted [formal carer support, home help], she didn’t want it. She felt that I should do everything and then I explained to her that I need to work. I told her that for her to eat it’s me, for the house to be warm it’s me, for her to go her [medical] appointments it’s me.

In other dyads, patients’ independent approach to decision-making was facilitated by the caregiver when the caregiver trusted the patient’s judgement and when the patient felt they were trusted by their caregiver:

She [patient] is a very strong-willed person. Every decision she has made is her own decision. There has been no input from me. I haven’t guided her in anyway … She knows if it is in her best interest. (CG9)

I had already said yes to any help that I could be offered … but [caregiver] would trust me to make a wise decision. (P9)

### Shared decision-making

We found that patients and caregivers also made decisions together. The majority of dyads (seven dyads) reported situations where they made decisions together. These dyads included one dyad where the patient also reported occasions where they made decisions on their own. Shared decision-making was perceived by patients and caregivers as the patient and caregiver making decisions as a team. For example, participant P11 expressed:

It is nice to have somebody when the decision has to be made, they [caregiver) will always put their two pence worth in and then decide together what is best.

Participants indicated that a key aspect of shared decision-making was the opportunity to revisit prior communications and collectively re-examine and as required, adjust their preferences for treatment and care. For instance, a patient and their caregiver articulated:

The decision is always back and forth between the two of us. It’s always trying to find the best possible outcome and the best possible solution. If it [chemotherapy] is beneficial and works, if I am in a good frame of mind, and if my body is good, I’ll go for it but if not, we sit down and say ‘now is not the right moment, not the time to do it.’ (P4)

If I feel he [patient] can do it [tolerate chemotherapy], I would say ‘yes. I think you can still go ahead.’ But when I feel this is becoming a torture, we also discuss it and I say ‘this is what I feel.’ (CG4)

Caregivers voiced that their role as advocators for the patient was important to ensure that the patient’s care needs were met. Advocating on behalf of the patient resulted in caregivers making decisions with the patient. For example, caregiver CG7 who advocated on behalf of his spouse (P7) shared:

She [patient] wants to stay at home as long as possible or if possible, to the very end. I have told her that we will look after her as best we can for as long as we can … It is an understanding we now have.

Indeed, patients’ reliance on their caregiver to help them manage their care prompted patients to make decisions with their caregiver about their treatment and care even when they perceived themselves to lead more in decision-making when compared to their caregiver:

I am probably the stronger [taking the lead] in terms of making decisions … [but] I couldn’t have done it [managed care] without him [caregiver] and for all the things that are involved along the way. (P3)

## Discussion

We have described dimensions of the patient–caregiver relationship that has been supportive as they navigated decision-making in specialist palliative care. The reciprocation of emotional support between the patient and caregiver found in this study was consistent with other studies in the field.^8,21^ To add to the evidence, we identified that emotional support between the patient and caregiver in palliative care featured when patients and caregivers recognized the need to accommodate to and/or resolve their differences in their preferences for patient treatment and care.

We identified two different approaches to decision-making in the data; independent decision-making and shared decision-making. Independent decision-making by patients was explained by their need to feel in control of their care and by their desire not to overly burden their caregiver. Patients' (with advanced illness) need to feel in control and the desire to alleviate the caregiver burden has been documented elsewhere.^23–25^ Our findings pertaining to patients’ wishes to be in control and to alleviate carer burden, extend our understanding of the reasons why some patients in specialist palliative care make decisions about their care without counsel from their caregiver.

We detected differences across dyads for patient-independent decision-making. While some caregivers trusted the patient in making decisions without their counsel, conflict arose for other caregivers particularly if decisions made by the patient resulted in them needing more assistance from the caregiver but which their caregiver struggled to provide. These findings add depth to our understanding of the complexity of patient and caregiver decision-making in palliative care. Patient independent decision-making can in some instances, be motivated by their desire not to burden their caregiver. However, an independent decision-making approach by the patient may also result in caregivers feeling more burdened in a caregiving role if they do not have the resources to accommodate patient preferences.

Shared decision-making between health care professionals, patients, and caregivers in palliative care is an important dimension of care for both research and practice.^14,26,27^ In our study, we found that shared decision-making between the patient and caregiver comprised a joint (re)-examination of and (re)-adjustment to each other’s preferences for and choices about treatment and care. Our findings also highlight the impact that caregiver advocacy for the patient^28,29^ and patient dependency on the caregiver^30,31^ in palliative care may have on the patient’s approach to decision-making. It is possible that some patients in specialist palliative care who are comfortable with making decisions without their caregiver may still make decisions with their caregiver because they become increasingly reliant on their caregiver as they advance in their illness.^6^ Of note, our sample comprised patient and caregiver dyads where most patients had advanced cancer. Caregivers of patients with advanced cancer in other studies have been identified as critical decision partners^32,33^ particularly when they were relied upon to manage patient care.^33^

Our findings have relevance for day-to-day clinical practice in palliative care for the following reasons. First, we have shown that the patient and caregiver in specialist palliative care can be of emotional support to one another not only when they make decisions together, but also when the patient makes decisions independent of their caregiver. Patient-independent decision-making may not necessarily undermine the quality of the patient and caregiver relationship when faced with decisions about treatment and supportive care. Hence, when discussing treatment and supportive care with patients and their caregivers, it is beneficial for health care professionals to focus on helping the patient and caregiver understand the reasons behind one another’s preferences and adjust to each other’s perspectives. Second, the findings point to the importance for health care professionals to remain cognizant of the differences between patients and caregivers in their preferences for patient care, and of how patients' increasing dependence on their caregiver might in some cases, limit patients to make decisions about their treatment and care that are consistent with their own preferences. Health care professionals in specialist palliative care have a responsibility to protect patient autonomy and at the same time, support the caregiver in their caregiving role. Strategies that can effectively incorporate the caregiver in shared decision-making, but which are fully tailored to guard patient autonomy, would be beneficial to develop for health care professionals in palliative care.

A primary strength of the study is that we identified in specialist palliative care, key supportive behaviors between the patient and caregiver in decision-making from the perspective of the patient and caregiver dyad. However, the study has limitations. First, all but one of the dyads comprised patients with cancer. Hence, the findings are primarily limited to cancer patient and caregiver dyadic decision-making. Second, the patient and caregiver in three of the 11 dyads were interviewed together which could have restricted them from communicating as openly as possible in their interview. Third, the sample size was small, and participants were recruited through one (albeit very large) regional specialist palliative care service in Ireland. The findings may not necessarily be representative of patient and caregiver dyads in specialist palliative care across other jurisdictions.

## Conclusion

Our study is one of few studies that has reported how patients and caregivers in specialist palliative care support one another in decision-making for patient treatment and care. We identified supportive behaviors among patient and caregiver dyads for both patient-independent decision-making and for patient and caregiver shared decision-making. The findings are particularly relevant for health care professionals in palliative care practice who initiate discussions about treatment and care options with the patient but also involve and/or accommodate caregivers in these discussions. Research focused specifically on why some patients involve caregivers in decision-making and others do not would be beneficial to conduct. A closer examination of the relationship between patient dependency on their caregiver and patient approach to decision-making is required.
