# Supplementary material for: How Do Patients and Caregivers in Advanced Illness Support One Another in Decision-Making for Patient Care? A Qualitative Interview Study of Patient and Caregiver Dyads in Specialist Palliative Care
Source: Palliat Med Rep. 2024 Sep 30;5(1):417–24. doi: 10.1089/pmr.2024.0047 (PMC11514579; doi:10.1089/pmr.2024.0047)
Supplement: Supplementary Data S1 [file pmr.2024.0047_supp_datas1.pdf]

Semi-structured interview guide (patient of dyad interviewed on their own)

1. Can you tell me about any important decisions that you have made about your treatment or care?

2. Can you tell me about how you go about discussing decisions with (carer's name) about your treatment or care?

3. When you made this / these decisions how did you decide what to do? Did you make the decision(s) on your own, or was it a joint decision with (carer's name)?
   If patient-only decision -
   Why didn't you involve (carer) in the decision?

4. When making decisions, what would you like from (carer's name)?

5. Making decisions about treatment or care can be difficult.
   Do you think it is important for caregivers to provide (emotional) support to patients when making these decisions? Why?
   Do you think it is important for patients to provide (emotional) support to caregivers when helping to make these decisions? Why?

6. Can you describe an example of when you gave (carer's name) support to help decide about your treatment or care?

7. Have there been times when you have disagreed about treatment or care decisions with (carer's name)?
   What was the decision, what was the disagreement, and how do you feel about the support you got from (carer's name) at the time?

8. How do you feel you are coping with your illness?
   If patient states that they are not coping -
   How do you think this impacts on your decision-making about your treatment or care?

9. Is there anything else you would like to say about what we have talked about?

Semi-structured interview guide (caregiver of dyad interviewed on their own)

1. Can you tell me about any important decisions that you have been involved in about (patient's name) treatment or care?

2. Can you tell me about how you go about discussing decisions with (patient's name) about their treatment or care?

3. When decisions were made, how did you decide what to do?
   If decision not made jointly (i.e., only by patient) -
   Why do you think (patient) didn't involve you in the decision?
   How did that make you feel?

4. When helping (patient's name) make decisions, what would you like from them?

5. Making decisions about treatment or care can be difficult.
   Do you think it is important for caregivers to provide (emotional) support to patients when making these decisions? Why?
   Do you think it is important for patients to provide (emotional) support to caregivers when helping to make these decisions? Why?

6. Can you describe an example of when you gave (patient's name) support to help decide about their treatment or care?

7. Have there been times when you have disagreed about treatment or care decisions with (patient's name)?
   What was the decision, what was the disagreement, and how do you feel about the support you got from (patient's name) at the time?

8. How do you feel you are coping with patient illness?
   If caregiver states that they are not coping -
   How do you think this impacts on your decision-making about (patient's name) treatment or care?

9. Is there anything else you would like to say about what we have talked about?

Semi-structured interview guide (patient and caregiver interviewed together)

1. Can you tell me about any important decisions that you have made about (patient) treatment or care? (P & CG)

2. Can you tell me about how you go about discussing with each other decisions about (patient) treatment or care? (P & CG)

3. When you made this / these decisions how did you decide what to do? Did you (patient) make the decision(s) on your own, or was it a joint decision?
   If patient-only decision: question to patient -
   Why didn't you involve (carer) in the decision?
   If patient-only decision: questions to carer -
   Why do you think (patient) didn't involve you in the decision?
   How did that make you feel?

4. When making decisions, what would you like from (carer's name)? (P)
   When helping (patient's name) make decisions, what would you like from them? (CG)

5. Making decisions about treatment or care can be difficult.
   Do you think it is important for caregivers to provide (emotional) support to patients when making these decisions? Why? (P & CG)
   Do you think it is important for patients to provide (emotional) support to caregivers when helping to make these decisions? Why? (P & CG)

6. Can you describe an example of when you gave (carer's name) support to help decide about your treatment or care? (P)
   Can you describe an example of when you gave (patient's name) support to help decide about their treatment or care? (CG)

7. Have there been times when you have disagreed about treatment or care decisions? (P & CG)
   What was the decision, what was the disagreement, and how do you feel about the support you got from one another at the time? (P & CG)

8. How do you feel you are coping with (patient) illness? (P & CG)
   If either person states that they are not coping
   How do you think this impacts on your decision-making about (patient) treatment or care?

9. Is there anything else you would like to say about what we have talked about?
